# Men perform comparably to women in a perspective taking task after administration of intranasal oxytocin but not after placebo

**DOI:** 10.3389/fnhum.2013.00197

**Published:** 2013-05-27

**Authors:** Angeliki Theodoridou, Angela C. Rowe, Christine Mohr

**Affiliations:** ^1^School of Experimental Psychology, University of BristolBristol, UK; ^2^Institute of Psychology, University of LausanneLausanne, Switzerland

**Keywords:** oxytocin, empathy, perspective taking, sex differences, self-report

## Abstract

Oxytocin (OT) is thought to play an important role in human interpersonal information processing and behavior. By inference, OT should facilitate empathic responding, i.e., the ability to feel for others and to take their perspective. In two independent double-blind, placebo-controlled between-subjects studies, we assessed the effect of intranasally administered OT on affective empathy and perspective taking, whilst also examining potential sex differences (e.g., women being more empathic than men). In study 1, we provided 96 participants (48 men) with an empathy scenario and recorded self-reports of empathic reactions to the scenario, while in study 2, a sample of 120 individuals (60 men) performed a computerized implicit perspective taking task. Whilst results from Study 1 showed no influence of OT on affective empathy, we found in Study 2 that OT exerted an effect on perspective taking ability in men. More specifically, men responded faster than women in the placebo group but they responded as slowly as women in the OT group. We conjecture that men in the OT group adopted a social perspective taking strategy, such as did women in both groups, but not men in the placebo group. On the basis of results across both studies, we suggest that self-report measures (such as used in Study 1) might be less sensitive to OT effects than more implicit measures of empathy such as that used in Study 2. If these assumptions are confirmed, one could infer that OT effects on empathic responses are more pronounced in men than women, and that any such effect is best studied using more implicit measures of empathy rather than explicit self-report measures.

## Introduction

Oxytocin (OT) is a highly conserved neuropeptide and an accumulation of its receptors are found in the amygdala (Loup et al., 1991). The amygdala is a structure that is part of the limbic system, associated with social behavior and emotion processing (Phelps and LeDoux, 2005), or more broadly with “relevance detection” (Sander et al., 2003). OT is involved in the regulation of the hypothalamic-pituitary-adrenal (HPA) axis, and thus affects processes such as birth and breast-feeding in females and sexual mating, attachment and bonding in both sexes (see Carmichael et al., 1987, 1994; Carter, 1992; Altemus et al., 1995; Meston et al., 2004; Vignozzi et al., 2008). OT is also proposed to have anxiolytic effects (Uvnäs-Moberg, 1997; Heinrichs et al., 2003). Thus, OT acts centrally and peripherally as a central neurotransmitter/neuromodulator and a peripheral hormone in both males and females (Carter, 1998; MacDonald and MacDonald, 2010).

In line with its anatomical and functional properties, OT is involved in human interpersonal information processing and behavior such as in enhancing prosocial judgments and behavior. For example, OT as compared to placebo administration (1) yielded higher trust in others (Kosfeld et al., 2005), (2) increased perceived attractiveness and trustworthiness of unfamiliar faces (Theodoridou et al., 2009), (3) increased charitable donations (Barraza et al., 2011), (4) promoted positive inferences about others' mental states (Domes et al., 2007), and (5) facilitated the identification of emotions regardless of valence (Lischke et al., 2012). Pointing to OT influences in clinical populations, individuals with autism benefited from OT administration by showing enhanced affective speech comprehension (Hollander et al., 2007), “mind-reading” (Guastella et al., 2010), and processing of social signals and social feedback (Andari et al., 2010). Likewise, in patients suffering from psychotic symptoms, OT administration improved performance in theory of mind tasks and perceptions of trustworthiness (Pedersen et al., 2011).

Given OT's role in interpersonal information processing and behavior, it can be assumed that it also plays a role in empathy, that is, the ability to understand another's emotional perspective and to be personally affected by it in a way that mirrors the feelings of the individual (Eisenberg and Miller, 1987). Empathy is crucial to successful interpersonal skills and relations (Miller and Eisenberg, 1988; Batson, 1991; Eisenberg et al., 2002). According to most models, empathy consists of at least two components (Gladstein, 1983; Mahrer et al., 1994; Kerem et al., 2001). The first component accounts for the cognitive effort involved in considering another's viewpoint (i.e., perspective taking), and the second concerns vicarious emotional affective empathy (Davis, 1983; Hoffman, 2000; Blair, 2005), herein referred to as “affective empathy.” Both are required for normal “empathic ability” (Davis, 1983; Duan and Hill, 1996; Cialdini et al., 1997) and are shown to be related (Thakkar et al., 2009; Mohr et al., 2010; Thakkar and Park, 2010; Gronholm et al., 2012) and modulated by individual difference variables and personal experiences (Mohr et al., 2010; Cooper and Mohr, 2012).

To date, examination of OT's role in empathy has been associated with two main pitfalls. Firstly, most studies have used self-report questionnaires, which lack accuracy and are prone to socially desirable responding Tierney and McCabe, 2001; Kämpfe et al., 2009; Gerdes et al., 2010; Taras et al., 2010. Secondly, research findings have been mixed, painting a rather unclear picture (Zak et al., 2007; Singer et al., 2008; Bartz et al., 2010; Hurlemann et al., 2010). On the one hand, studies show that OT administration enhances self-reported emotional (but not cognitive) empathy (Hurlemann et al., 2010) as measured by the Multifaceted Empathy Test (MET; Dziobek et al., 2008) as well as empathic accuracy (Bartz et al., 2010), that is, the ability to accurately rate others' feelings when they narrate emotional events, in particular in listeners that are not socially proficient. Also, OT as compared to placebo administration enhanced perspective taking ability and generosity toward others in an economic game; i.e., more money was transferred to partners after having imagined their perspective and considered their reaction to an offer (Zak et al., 2007). On the other hand, OT versus placebo administration exerted a null effect on emotional empathic responses to a romantic partner's pain, i.e., on self-reported unpleasantness ratings when considering the partner's experience of painful hand stimulation (Singer et al., 2008). These inconsistent findings may either be due to OT and empathy being unrelated, or problems with the self-report measurement of empathy. We here consider these possibilities.

**Figure 1:**
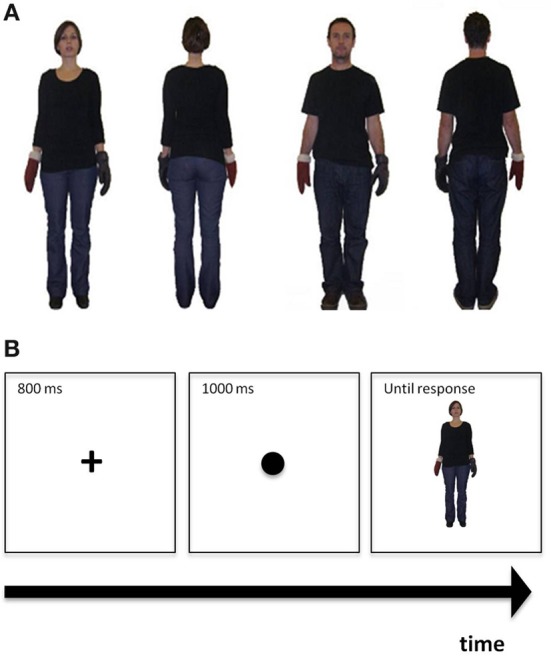
**(A)** Examples of figures used in the perspective taking task. From left to right: The first two frames illustrate front- and back- facing female figures, while the remaining two frames depict front- and back- facing male figures. **(B)** Demonstration of the perspective taking task procedure: The cross is presented first, followed by the coloured dot, followed by the figure.

In two independent studies, we used comparable double-blind placebo-controlled between-subject designs to assess healthy individuals' empathy as a function of nasal OT administration. In study 1, we provided participants with a vignette in which a person's unfortunate plight was described. Participants rated their empathic feelings toward the individual (see e.g., Coke et al., 1978; Batson et al., 1989; Mikulincer et al., 2001); thereby directly linking the self-reported empathic response to an individual's plight. In study 2, we used a more implicit strategy by assessing reaction times for perspective taking in a computerized task. In this task, participants see back-facing and front-facing human figures sequentially on the computer screen and have to match the own perspective with the one of the figure (e.g., Mohr et al., 2010; Thakkar and Park, 2010; Gardner et al., 2012). Matching the own body position with that of a front-facing figure is cognitively more challenging than matching it with a back-facing figure as reflected in enhanced reaction times (see Figure 1A). This task has been used to examine various questions on cognitive functioning such as those underpinning different forms of mental rotation (Ratcliff, 1979; Zacks et al., 1999), cognitive correlates of out-of-body experiences (Blanke et al., 2005; Easton et al., 2009; Braithwaite et al., 2011) and schizotypy (Mohr et al., 2006; Easton et al., 2009), to evaluate learning (Bailey et al., 2007), and spatial compatibility effects (Gardner and Potts, 2011). Empirical evidence showed that at least part of task performance variance is modulated by empathy. For instance, in this 3^rd^ person perspective taking task (3PP-task) increasing self-reported empathy scores are negatively correlated with response speed (Thakkar et al., 2009) in women but also positively correlated with greater accuracy (Thakkar and Park, 2010) and reaction times (Mohr et al., 2010), in particular for individuals reportedly using a social rather than a spatial perspective taking strategy (Gronholm et al., 2012). Thus, if OT enhances empathy, irrespective of the assessment format chosen, we would expect that increased OT availability enhances individuals' empathy in both studies, leading to higher empathic concern ratings in study 1 and potentially faster reaction times in study 2. Yet, if explicit, self-report measures of empathy bias desirable responding, the effect of OT might not be observed in study 1, with the 3PP task in study 2 producing more pertinent results, at least statistically.

In addition to these hypotheses, we considered the role of participant sex. Some studies of OT effects support an enhancing role of OT for interpersonal behavioral responses in both sexes (for evidence of absence of sex-dimorphic effects of OT see Ditzen et al., 2009; Theodoridou et al., 2009, 2011; Alvares et al., 2010; Rockliff et al., 2011), but others yield direct evidence for potential sex-specific effects of this neuropeptide Fischer-Shofty et al., 2013; Theodoridou et al., 2013; for a recent review on the role of sex in individual responses to OT see MacDonald, 2012). It is worth noting that studies that have reported effects of OT specifically on empathy and/or perspective taking either recruited only men (Zak et al., 2007; Bartz et al., 2010) or found that OT selectively affected men (Hurlemann et al., 2010). In light of the above inconsistent evidence (Ditzen et al., 2009; Hurlemann et al., 2010), we considered the possibility that OT might exert sex-specific effects on empathy and perspective taking and tested an equal number of women and men.

## Materials and methods

### Procedure common to both studies

In both studies, we conducted two sessions: a baseline session performed by participants at home and a laboratory session for which participants came to the University. Before participation in either session we obtained written informed consent from each participant. We only tested participants who met our inclusion criteria: being a fluent English speaker, not having consumed any medication, or having any other medical reason why they should not receive OT. In the case of female participants, they could not be pregnant, or if post-birth, should not be breastfeeding. The research protocol in both studies was approved by the Faculty of Science Human Research Ethics Committee at the University of Bristol. We recruited participants through poster advertisement in and around university buildings and sent emails to various departments and posted on the university's jobs website. In the baseline session, participants provided demographic information (e.g., age, sex) and filled in self-report questionnaires such as the Major Depression Inventory (MDI; Bech, 1997); results from these measures can be found in Theodoridou et al. (2009) and Theodoridou et al. (2013). Participants sent the completed questionnaires to the experimenter.

In the laboratory session at the local university, approximately one week later, participants were tested individually. They were instructed to abstain from alcohol, caffeine, and nicotine for 24 h before testing and from food and drink (except water) for 2 h before testing. When they arrived in the laboratory, each participant signed an informed consent form. The session lasted up to 2 h, with the actual task battery being assessed in the first 60 min after the waiting period, and the remaining time being used to guard for potential side effects. At the very beginning, participants were told that they would first receive a small dose of OT or a near identical looking and smelling placebo before being tested in various tasks. Information was also given about possible side effects associated with OT administration and participants were informed that they had the right to withdraw from the study at any time. In a double-blind procedure, participants were randomly assigned to self-administer a small intranasal dose of either 24 IU OT (Syntocinon Spray, Novartis, 3 puffs per nostril, each puff containing 4 IU OT), or placebo (containing the same ingredients, but OT, to the OT nasal spray). After a waiting period of 25–30 min, participants completed the task battery including the empathy vignette task (Study 1) and the 3PP-task (Study 2). Tasks were presented in two blocks, randomized in order. In study 1, the empathy scenario task was administered either 35 or 55 min after drug administration. In study 2, the 3PP-task was completed either 35 or 60 min after drug administration (see Theodoridou et al., 2009, 2011, 2013 for additional results from these studies). This time window is likely sensitive to OT effects; Gossen et al. (2012) showed that OT reaches its peak plasma level at approximately 30 min after a dose of 26 IU intranasal OT. Also note that the present protocol has led to established methods and findings in our laboratory before (see Theodoridou et al., 2009, 2011, 2013).

In the laboratory sessions, we also assessed current mood, wakefulness, and calmness with the short form of the Multidimensional Mood State Questionnaire (Steyer et al., 1997). This 6-point scale consists of 15 items with answers ranging from “definitely not” to “extremely.” In Study 1, this measure was completed once, whereas in Study 2, it was completed twice, immediately before drug administration and immediately before testing. After testing, participants were instructed to guess the substance they had received^1^. Finally, participants were debriefed and offered either a monetary reward of £15 or experimental credit (or chocolate in a few instances).

### Study 1

#### Participants

Of the 96 participants (mainly students, mean age: 21.4 years, age range: 18–40 years), 51 (25 males) received OT, and 45 received placebo (23 males). As reported in our previous study (Theodoridou et al., 2009), any drug effect, sex effect, or interaction cannot be explained by participants' current affect, wakefulness or calmness. Likewise, independent samples *t*-tests showed no significant differences between drug groups in depression and trait anxiety (see Theodoridou et al., 2011 for details).

#### Empathy task

Emotional reactions to another person's plight were assessed using a procedure similar to that employed previously (e.g., Coke et al., 1978; Batson et al., 1989; Mikulincer et al., 2001). More precisely, the experimenter read out a brief story about the plight of a university student who had recently lost her parents in a car accident. The full story read as follows: “*Anna is 21 years old studying, on a full-time basis at the University of Bristol. A month ago her parents and older sister got killed in a car accident. At the moment she is desperately trying to take care of her surviving younger brother and sister while trying to finish her last year of BSc studies. If she does not complete her degree, she will not be able to earn enough money to support her brother and sister and will have to put them up for adoption. What is more, Anna has no relatives that can help her out*.” Immediately afterwards, the experimenter read out 10 adjectives (taken from Batson et al., 1989; see below), each of which participants verbally rated on a 7-point visual analog scale according to how they felt while listening to the story (1—not at all felt, 7—very strongly felt). Participants were asked to bear in mind that the student was a mere acquaintance to them. We stressed this point because we wished to examine the effect of OT on prosocial behavior toward non-intimate others to avoid ceiling effects due to emotional closeness to the main character of the story. A digital voice recorder (Olympus; VN-2100PC) was used to record responses in this task.

Half of the adjectives tap on empathic concern (i.e., other-oriented emotional empathy at the plight of others): *sympathetic*, *soft-hearted*, *compassionate, tender, moved*, and the other half tap on feelings of personal distress (i.e., self-oriented emotional reactions at the plight of others): *alarmed*, *grieved, distressed, upset, and disturbed*. We calculated mean scores for empathic concern responses and personal distress responses, separately (range of scores 1–7 with higher scores reflecting greater empathic concern and personal distress, respectively) to account for the possibility that these two dimensions are differently influenced by OT and/or sex. For instance, OT might increase empathic concern for a person in need and/or attenuate feelings of personal distress (Batson et al., 1987). OT might act anxiolytically (Carter et al., 2001; Heinrichs et al., 2001, 2003; Cardoso et al., 2012; de Oliveira et al., 2012; for reviews see Meyer-Lindenberg et al., 2011; Striepens et al., 2011; MacDonald and Feifel, 2012) decreasing discomfort and concern for one's own self.

### Study 2

#### Participants

Of the 120 participants (mainly students, mean age: 22.4 years, age range: 18–44 years), 60 (30 males) received OT, and 60 received placebo (30 males). As in study 1 (Theodoridou et al., 2009), we observed no influence of drug and/or sex on participants' current affect, wakefulness, or calmness. In more detail, following Domes et al.'s (2007) procedure, we calculated difference scores on the different mood measures affect, wakefulness and calmness for time 1 (pre drug administration) and time 2 (30 min post drug administration) by subtracting Mood at time 2 from Mood at Time 1. Thus, positive values reflect better mood at time 1 and negative values reflect better mood at time 2. We performed a multivariate ANOVA (MANOVA) on these difference scores with drug (OT, placebo) and participant sex (male, female) as between-subjects variables. There was no effect of drug on change in affect, *F*_(1, 116)_ = 0.02, *p* = 0.90, wakefulness, *F*_(1, 116)_ = 0.04, *p* = 0.85, and calmness, *F*_(1, 116)_ = 0.06, *p* = 0.80. Similarly, no effect of participant sex was found on change in affect, *F*_(1, 116)_ = 0.10, *p* = 0.75, change in wakefulness, *F*_(1, 116)_ = 0.02, *p* = 0.90, and change in calmness, *F*_(1, 116)_ = 1.71, *p* = 0.19, and no significant drug × participant sex interactions (all *p*s > 0.1).

#### Perspective taking task

***Picture preparation.*** To make figures more realistic, we here refrained from using schematic drawings used before (Mohr et al., 2010, in press; Cooper and Mohr, 2012) and took photographs of an adult man and an adult woman of approximately the same height instead, both dressed in jeans and black T-shirts (see also Thakkar et al., 2009; Thakkar and Park, 2010). The photographs showed these adult models standing upright with the arms slightly outstretched to the right and left (see Figure 1A). Each of them was photographed in this same position from the front and the back. For each picture taken, the models were always wearing a black glove on the one hand and a brown glove on the other hand. The side of the black glove was once on the right and once on the left for both front-facing and back-facing positions. This counterbalancing resulted in eight possible photographs that were all of the same size (237 × 239 pixels), cropped and set against a white background (see Figure 1A for representative examples). For single trials, a centrally placed fixation cross appeared first for 800 ms followed by a centrally presented dot (diameter = 1.5 cm) for 1000 ms that was black in half of the trials and light brown in the remaining trials. After the disappearance of the dot, one of the pictures was presented centrally (visual angle ≈ 5° width × 6.1° height) until a response was provided, that is, the task was self-paced.

***Task procedure.*** First, participants received the written instruction that the following task would assess their empathic ability. The exact instruction was as follows: *“This is a test of your ability to see the world from another person's perspective. Performance on this test reflects empathetic ability. Empathy is a social skill that is defined as being able to identify with, and understand what another person is perceiving, and to respond appropriately.”* This instruction was based on previously used instructions (Massa et al., 2005) and was included to ensure that the task was introduced as a test of empathic abilities. Participants then received both a written and a verbal instruction to imagine being in the other person's shoes. Specifically, the instruction was: *“Imagine you are in the person's body position. If the coloured circle is black, indicate which hand the black glove is on. If the coloured circle is brown, indicate which hand the brown glove is on. If the glove would be on your own left hand, press key ‘1.’ If the glove would be on your own right hand, press key ‘5.’ Please respond as quickly and accurately as possible, but always aim to take the other person's perspective first.”* Following task instructions, a slide with the demonstration of the task procedure was presented for 6000 ms (see Figure 1B). Each picture was presented 10 times resulting in 80 experimental trials. The task was preceded by eight practice trials. We assessed the number of correct responses and the reaction times for correct responses.

## Data analysis

In Study 1, data from one participant was excluded because this person responded especially slowly (average response time: 18 s). We also excluded scores (ratings) that were two standard deviations above or below the mean (1.51% of the data). A MANOVA test was carried out on mean empathic concern ratings and mean personal distress ratings, with drug (OT, Placebo) and participant sex (male, female) as between-subjects variables. One-sample *t*-tests were also performed on the key dependent variables in study 1, namely, mean empathic concern and personal distress ratings against the chance value of 3.5. Further one-sample *t*-tests were carried out, on the empathic concern ratings and on the personal distress ratings separately for the sexes in each drug group.

In Study 2, reaction times shorter than 200 ms and longer than 5000 ms were considered to be outliers and were dropped (Harris et al., 2002; Mohr et al., 2010). Incorrect trials were also discarded. The data of five participants were discarded because their error rates (ranging from 27.5% to 48.75%) were more than two standard deviations above the mean (Mean error rate = 7.84%, *SD* = 8.24). A mixed model ANOVA was carried out on the mean RT data, with drug (OT, placebo) and participant sex (male, female) as between-subjects variables, and target sex (male, female), position (front, back) as repeated measures. No statistical analyses were performed on accuracy data as the average error rate (%) after removal of the five outlying cases was very low (Total Mean = 6.59%; OT Mean: 6.68%; Placebo Mean: 6.49%). Pairwise *post-hoc* comparisons were based on Newman-Keuls tests. In both studies, we performed univariate ANOVA tests to examine potential age differences between the two drug groups and the two sex groups. The alpha level for all the statistical test results reported henceforth was set to 5% (0.05).

## Results

### Study 1: OT and ratings of adjectives subsequent to an empathy scenario

#### Participants

The ANOVA on age showed no significant main effects [drug: *F*_(1, 92)_ = 0.33, *p* = 0.57, participant sex: *F*_(1, 92)_ = 0.003, *p* = 0.96] and no significant interaction, *F*_(1, 92)_ = 0.01, *p* = 0.91.

#### Adjective ratings

The MANOVA showed that OT (vs. placebo) had no effect on empathic concern, *F*_(1, 90)_ = 0.16, *p* = 0.69, or personal distress ratings, *F*_(1, 90)_ = 0, *p* = 0.99. A significant main effect of participant sex was found for empathic concern ratings, *F*_(1, 90)_ = 4.18, *p* = 0.04, with females self-reporting greater empathic concern (*M* = 5.30, *SD* = 0.99) than males (*M* = 4.93, *SD* = 0.77). No effect of sex was found on personal distress scores, *F*_(1, 90)_ = 1.91, *p* = 0.17. We did not observe any significant interactions [largest interaction effect: *F*_(1, 90)_ = 1.41, *p* = 0.24]^2^.

When comparing the two adjective rating scores against chance level (3.5), the one-sample *t*-tests showed that the mean empathic concern rating (*M* = 5.12; *SD* = 0.90) was significantly higher than chance level, *t*_(93)_ = 17.38, *p* < 0.001, as was the mean personal distress rating (*M* = 3.83; *SD* = 1.27), *t*_(93)_ = 2.52, *p* = 0.01. The same conclusion could be drawn when performing the same comparisons for the two sexes in each drug group, separately (see Table 1 for detailed results). The opposite conclusion could be drawn when performing the same comparisons for mean personal distress ratings, i.e., the mean adjective ratings scores were not different from chance level, apart from higher scores in female participants in the placebo group (see Table 1).

**Table 1:** **Mean empathic concern ratings and their difference from chance level (3.5) for the two sexes in each drug group**.

		**Empathic concern**	***t* (*p*-value)**	**Personal distress**	***t* (*p*-value)**
OT	M	4.93 (0.64)	10.93 (<0.001)	3.80 (1.01)	1.47 (0.15)
	F	5.23 (0.89)	9.82 (<0.001)	3.86 (1.46)	1.24 (0.23)
P	M	4.93 (0.91)	7.39 (<0.001)	3.49 (1.18)	−0.04 (0.97)
	F	5.39 (1.10)	8.03 (<0.001)	4.16 (1.35)	2.30 (0.03)

Notes: standard deviations are in parentheses; OT, oxytocin; P, placebo; M, Male; F, Female; OT Male df = 23; P Male df = 21; OT Female df = 25; P Female df = 21.

### Study 2: OT and perspective taking

#### Participants

The ANOVA on age showed no significant main effects [drug: *F*_(1, 116)_ = 0.87, *p* = 0.35, participant sex: *F*_(1, 116)_ = 1.11, *p* = 0.29] and no significant interaction, *F*_(1, 116)_ = 0.02, *p* = 0.89.

#### Reaction time analysis for the 3PP-task

The ANOVA on mean reaction times for correct decisions showed a significant main effect of figure position, *F*_(1, 111)_ = 124.03, *p* < 0.001, with front-facing figures eliciting longer reaction times (*M* = 1122.53, *SD* = 346.86) than back-facing figures (*M* = 913.93, *SD* = 255.64). In addition, the main effect of figure's sex, *F*_(1, 111)_ = 31.68, *p* < 0.001, indicated that participants responded faster to female figures (*M* = 994.69, *SD* = 271.36) than to male figures (*M* = 1040.09, *SD* = 304.05). The main effects of drug, *F*_(1, 111)_ = 1.8, *p* = 0.18, and participant sex, *F*_(1, 111)_ = 1.35, *p* = 0.25, were both not significant^3^.

**Figure 2:**
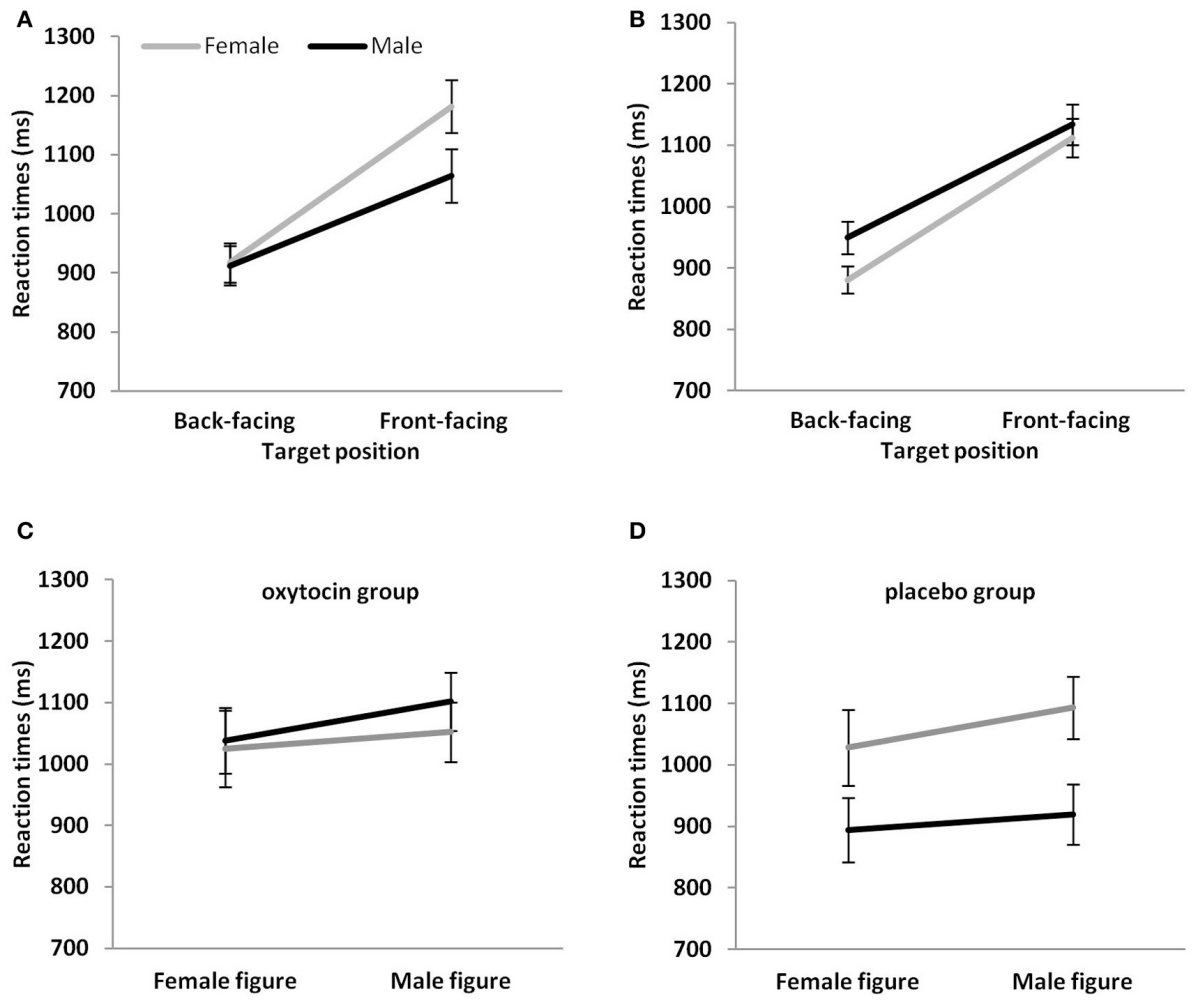
**Mean reaction times (A) of male and female participants to target figures in the back- and front-facing position, **(B)** to male and female target figures in the back- and front-facing position, **(C)** of male and female participants to target figures in the OT group and **(D)** the placebo group.** Vertical bars indicate standard errors.

We found significant 2-way interactions between figure position and participant sex, *F*_(1, 111)_ = 9.03, *p* = 0.003, and figure position and target sex, *F*_(1, 111)_ = 10.3, *p* = 0.004, and a significant 3-way interaction between drug, target sex, and participant sex, *F*_(1, 111)_ = 5.7, *p* = 0.02. *Post-hoc* comparisons on these significant interactions showed for the figure position by participant sex interaction that male participants had faster reaction times than female participants for the front-facing condition (*p* = 0.04) (see Figure 2A). The same comparison for the back-facing condition was not significant (*p* = 0.92). Moreover, reaction times were significantly faster in the back- than front-facing condition for both male (*p* < 0.001) and female participants (*p* < 0.001). *Post-hoc* comparisons on the figure position by target sex interaction showed that female figures were responded to faster than male figures in the back-facing condition (*p* < 0.001) and front-facing condition (*p* = 0.04) (see Figure 2B). In addition, reaction times for back-facing figures were significantly faster than for front-facing ones for both male and female targets (all *p*s < 0.001).

Finally, to further elucidate the significant drug, target sex, and participant sex interaction, we performed 2-way ANOVAs, for the OT and the placebo group separately, with figure sex as a repeated measure and sex as a between subject-factor. The ANOVA for the OT group only showed the significant main effect on figure sex, *F*_(1, 56)_ = 11.88, *p* = 0.001, i.e., that reaction times were faster for female than male figures. The main effect of sex, *F*_(1, 56)_ = 0.14, *p* = 0.71, and the interaction, *F*_(1, 56)_ = 1.96, *p* = 0.17, were both not significant (Figure 2C). The ANOVA for the placebo group, however, showed in addition to the main effect of figure sex, *F*_(1, 54)_ = 24.58, *p* < 0.001 (male figures > female figures), a main effect of participant sex, *F*_(1, 54)_ = 6.34, *p* = 0.01. Male participants responded faster than female participants (Figure 2D). Finally, the interaction was significant, *F*_(1, 54)_ = 4.25, *p* = 0.04. *Post-hoc* comparisons showed that reaction times were faster for female than male figures in female (*p* < 0.001) and male (*p* = 0.045) participants. Moreover, male as compared to female participants showed a significant reaction time advantage for male figures (*p* = 0.02) that was only a statistical trend for female figures (*p* = 0.08).

## Discussion

In two independent double-blind, placebo-controlled between-subject design studies we investigated whether the consumption of a single dose of OT affected the ability to empathize with another individual's unfortunate plight (Study 1) and the ability to mentally take the perspective of another person (Study 2). In study 1, we provided participants with a scenario designed to elicit empathy [modified version used by Coke et al. (1978); Batson et al. (1989)] and assessed two levels of self-reported empathy (empathic concern and personal distress) by having participants rate empathy relevant adjectives. In study 2, we recorded reaction times in a computerized 3PP-task to assess empathy more implicitly (e.g., Thakkar et al., 2009; Mohr et al., 2010, in press) and to also avoid the response biases often associated with self-report measures (Kämpfe et al., 2009). Given that OT has been previously shown to enhance interpersonal processes and behavior (for reviews see MacDonald and MacDonald, 2010; Bartz et al., 2011; Guastella and MacLeod, 2012); including empathy (Hurlemann et al., 2010) and related phenomena such as “mind-reading” (Domes et al., 2007; Guastella et al., 2010), we hypothesized that OT as compared to placebo administration would result in enhanced empathic responses (study 1) and speeded perspective taking (study 2). We also considered that this enhancement might be more prominent in study 2, because response biases associated with self-report measures might override the OT effect in study 1. Our main findings were that (1) the adjective ratings in study 1 (empathy scenario) did not reveal higher empathic responses in the OT as compared to the placebo group, and (2) sex differences in the 3PP-task (male over female participant advantage, in particular for male figures) observed in the placebo group were absent in the OT group. Before discussing these results and additional task findings, we would like to mention that neither study 1 nor study 2 found effects of OT administration on affect, wakefulness and calmness, indicating that the above reported behavioral effects of OT could not be attributed to or mediated by changes in mood. These findings are in agreement with previous studies that have reported null effects of OT on mood (Kosfeld et al., 2005; Domes et al., 2007; Fischer-Shofty et al., 2010; Lischke et al., 2011).

### OT and empathy ratings when exposed to the plight of an unknown person

In study 1, the OT and placebo group provided comparable adjective ratings after hearing about an unknown person's plight. By inference the two groups reported comparable empathic reactions and personal distress when rating these adjectives. Contrary to previous findings supporting the role of OT in prosocial and affiliative behavior (Kosfeld et al., 2005; Theodoridou et al., 2009; Barraza et al., 2011), our results suggest that OT may not play a role in the experience of affective empathy. However, given that to be empathetic is a socially desirable trait and that participants spoke aloud their answers to the experimenter, we can assume that our methodology was prone to self-report biases (e.g., see Tierney and McCabe, 2001; Kämpfe et al., 2009; Gerdes et al., 2010; Taras et al., 2010). Our findings indicate that participants seemed inclined to provide high scores in these ratings: the average empathic concern rating was 5.12, thus, biased toward the highest possible score of seven. Our results show that this mean rating was significantly different to chance level 3.5 (i.e., mid-scale point), and that our female participants' self-reports of empathy deviated more strongly from this chance level than did male participants' self-reports. What is more, the rating of 5.12 appears high compared to values obtained in previous studies using similar (but not identical) designs, and in which seven was also the highest possible value [e.g., see Batson et al., 1988, studies 1 and 3 (3.81 and 3.88, respectively); Batson et al., 1989, study 2 (4.44 and 5.79)]. Consistent with sex differences observed by Batson et al. (1988, 1989), women scored higher in empathic concern than men (see also Baron-Cohen and Wheelwright, 2004; Hurlemann et al., 2010). As for personal distress ratings, the mean value here was lower than the value in a comparable study (e.g., in Batson et al., 1988, study 2). Given the overall caution with regard to self-report measures in this domain (Baron-Cohen and Wheelwright, 2004; Kämpfe et al., 2009), we suggest that empathic concern adjectives might tap onto sex stereotypes regarding emotions, i.e., women being more caring and higher in sympathy than men (Plant et al., 2000). By inference, such stereotypes may trigger stronger response biases and desirable responding than personal distress adjectives.

Importantly, our results show no effect of drug administration on personal distress or empathic concern. Therefore, our findings fail to provide evidence for an anxiolytic and prosocial effect of OT, respectively, in the context of affective responses to an individual's plight. It is worth noting that in line with previous relevant studies (Batson et al., 1988, 1989; Mikulincer et al., 2001) we only tested affective responses to a *woman*'*s* plight. Future studies should balance out for possible sex-specific effects and include a male target, although a faster reaction toward women is generally likely (see Lewin and Herlitz, 2002; Cellerino et al., 2004; Mohr et al., 2010) in line with our finding of speeded responses to female figures in study 2.

To further understand the influence of sex on the link between OT effects on empathic abilities we tested an equal number of women and men. Studies in which individuals were provided with audio tapes narrating the plight of a needy person showed that women reported higher levels of empathy than men for the needy person (Batson et al., 1988, studies 1 and 3), and that reports of empathy for the needy person were relatively high (Batson et al., 1988, studies 1 and 3; Batson et al., 1989, study 2). Such sex differences in self-reports of empathy have often been documented, but could reflect a female tendency toward more socially appropriate responding relative to men (Eisenberg and Miller, 1987). In our study we did indeed observe that females' self-reports of empathy deviated more strongly from chance level than did males self-reports. However, no differential effect of OT was found in men's and women's self-reported empathy.

### OT and reaction times in a computerized 3PP-task

In study 2, we found that speed of response in the drug groups interacted with figure sex and participant sex. We observed that sex differences in the placebo group were absent in the OT group. More precisely, in both drug groups, we found that people responded faster to female than male figures. In the placebo group, we additionally observed that men responded faster than women, and that this sex difference was statistically significant for male figures and a statistical trend for female figures. Given that these sex differences are absent in the OT group, we infer that OT might have sex-dimorphic effects on this measure of perspective taking ability, an inference that is in line with a recent review by MacDonald (2012). The observation that male participants in the OT group responded as slowly as female participants in the OT group could further indicate that men adopted a comparable perspective taking strategy to the one used by women. This conjecture assumes that the women in our study, irrespective of whether they have received OT or placebo, adopt a relatively more time-consuming social perspective taking strategy when completing the 3PP-task as compared to the men in the placebo group. Evidence for this suggestion comes from research indicating that women experience greater rotational costs for front-facing figures (reflected in increased reaction times and decreased accuracy) than men when performing a 3PP-task (Mohr et al., 2010). In addition, further studies on computerized perspective taking ability indicate that social strategies might be more prevalent in women and object-based spatial strategies more prevalent in men, and that women find the 3PP-task more effortful, as reflected in lower accuracy rates (Kaiser et al., 2008). The link between faster reaction times and self-reported empathy seems to be most evident for women (Mohr et al., 2010), while no role of sex is reported in two further studies (Thakkar and Park, 2010; Gardner et al., 2012). Finally, studies using a similar (but not identical) perspective taking task to ours showed that women with high affective empathy scores are slower on perspective taking (Thakkar et al., 2009; but also see Thakkar and Park, 2010).

If OT fosters social perspective taking in men, we could expect in future studies that a higher than normal OT availability may facilitate men's tendency to step into another person's shoes, a fundamental component of empathy (e.g., see Kaiser et al., 2008; Thakkar et al., 2009; Gardner et al., 2012). The present conclusion is relevant to empathy and OT researchers as it suggests that OT's beneficial effects might not be general but nuanced (Bartz et al., 2011), i.e., affecting men more strongly than women, at least in the context of social perspective taking. Alternatively, such beneficial OT effects might be evident in those who are less socially proficient, which men are thought to be (see also Bartz et al., 2010).

We suggest that the above findings on OT effects in men are not an artifact of overall or aberrant performance in our study population, because we replicated previous behavioral findings using slightly modified versions of the current 3PP-task. Firstly, reaction times were faster for back-facing than front-facing pictures (see also e.g., Arzy et al., 2007; Mohr et al., 2010, in press; Thakkar and Park, 2010; Cooper and Mohr, 2012) indicating that participants performed mental transformations. This finding is in line with previous reports regarding the mental rotation of objects (Shepard and Metzler, 1971; Wohlschläger and Wohlschläger, 1998), body parts (Cooper and Shepard, 1975; Parsons, 1987; Bonda et al., 1995; Petit et al., 2003; Seurinck et al., 2004), and perspective taking tasks (e.g., Kaiser et al., 2008; Rilea, 2008) which report longer reaction times when the position of a stimulus (or own current body position) does not match the position of the target stimulus. Moreover, mental perspective transformations for female figures were faster than those for male figures, an advantage that was specific to back-facing figures, supporting previous observations (see Mohr et al., 2010).

## Conclusions and possibilities

Two studies examined the differential effects of OT on empathic responses, once using self-report ratings after having heard the story of an unknown person's unfortunate plight (study 1) and once using reaction times in a computerized 3PP-task (study 2). The major findings were that while OT as compared to placebo administration did not enhance self-reported empathic concern toward others (study 1), it showed that a male over female advantage in the 3PP-task that was evident in the placebo group was absent in the OT group (study 2). This finding is suggestive of a potential strategy change (purportedly more social than spatial performance strategy, see rationale in more detail above) in men after consuming OT as compared to placebo. Such a facilitation of social perspective taking might already be present in women, regardless of which of the two drugs were consumed. Thus, additional OT availability might affect men but not women in the 3PP-task. This conjecture, if supported in the future, might be relevant to sub-populations low in appropriate social abilities, such as individuals with alexithymia, social anxiety disorder, and schizophrenia (Caldwell et al., 2009; Guastella et al., 2009b; Bartz et al., 2010; Feifel et al., 2010; Rubin et al., 2010; Luminet et al., 2011; Pedersen et al., 2011; Hall et al., 2012) as it has already been shown in autism spectrum disorders (Andari et al., 2010; Guastella et al., 2010).

Given the conjectural nature of our conclusions, future studies should verify the strategy participants employ in the 3PP-task, e.g., examine whether women are slower because they use a social perspective strategy while men (at least without pharmacologically enhanced OT availabilities) use a spatial perspective taking strategy (Gardner et al., 2012; but also see Gronholm et al., 2012). Indeed, one could also reason that OT administration hindered a spatial strategy that might have been favored by men, without having to necessarily facilitate a social strategy. While this possibility cannot be excluded, we consider our initial explanation to be more likely because men and women seemingly used different strategies in perspective taking including mental rotation (e.g., Weiss et al., 2003; Kaiser et al., 2008), and women with higher self-reported empathy were found to slow down in perspective taking, presumably because they are using a social strategy that is also more time-consuming (Thakkar et al., 2009). However, it should be noted here that other studies have shown better perspective taking to be linked to faster reaction times (e.g., Mohr et al., 2010; Thakkar and Park, 2010). Given that the above studies assessed empathy via self-report and that such approaches are problematic, we propose that future studies would benefit from using more objective measures of empathy (e.g., actual behavioral observations and/or facial mimicry) to examine whether higher empathy is associated with slower or faster reaction times.

Another potential explanation of the above finding is that OT administration generally slows men down. It should be noted that whilst OT administration has been shown to slow men down in contexts other than spatial processing, such as during approach-avoidance motor responses to emotional faces (Theodoridou et al., 2013) and identification of fearful faces (Di Simplicio et al., 2009), it has not been found to have a slowing effect on approach-avoidance motor responses to non-social stimuli (Theodoridou et al., 2013), early processing (i.e., detection speed) of angry, and happy faces (Guastella et al., 2009a) and recognition of emotional faces (Fischer-Shofty et al., 2010). Therefore, it seems unlikely that OT administration generally slows men's speed of response.

Importantly, the sex differences and the interaction with drug group were observed in a task that was introduced as a task that assesses empathy. Future research could introduce the task as one that assesses mental rotation (see also Massa et al., 2005 for instruction effects), to test whether drug effects are influenced by such context effects. This would provide us with some further insight regarding task expectancies, and their interaction with drug treatment. In any case, we suggest that the 3PP-task was powerful at showing drug by sex interactions because such reaction time measures are difficult to manipulate/see through relative to a self-report task, thereby rendering our participants less able to guess our specific experimental hypotheses. This advantage might also explain why no drug effect or drug by sex interaction was observed in study 1, in which participants are actively asked to rate their feelings toward an unfortunate person in the presence of the experimenter. We suggest that response biases such as social desirability and stereotyping come strongly into play when using such paradigms, resulting in artificially elevated scores and commonly observed sex differences (women having higher scores than men) when using self-report measures (see for example Massa et al., 2005; Wraga et al., 2007). Furthermore, this is unlikely to be the case when using implicit measures such as the current perspective taking task (Mohr et al., 2010, in press; Thakkar and Park, 2010; Gardner et al., 2012). Thus, our findings indicate that more implicit measures such as the current 3PP-task might be better suited to assessing empathy, and that any effects of empathy-related processes whether cognitive or neurochemical (such as OT in the present case) might emerge more consistently when using measures that are less response bias prone.

### Conflict of interest statement

The authors declare that the research was conducted in the absence of any commercial or financial relationships that could be construed as a potential conflict of interest.

## Footnotes

^1^As reported in previous studies on results obtained from this population, participants could not predict the treatment they received (see Theodoridou et al., 2009, 2013).

^2^No effect of task order (task administered 35 min vs. 55 min after drug intake) was found when it was added as a covariate to the MANOVA (*p* > 0.05).

^3^When task order (task administered 35 min after drug intake, task administered 60 min after drug intake) was added to the above ANOVA as a covariate no effects involving task order were found (all *p*s > 0.05).
